# Road expansion risk predicts future hotspots of tropical deforestation

**DOI:** 10.1073/pnas.2502426122

**Published:** 2025-12-22

**Authors:** Jayden E. Engert, Carlos M. Souza, Fritz Kleinschroth, F. Yoko Ishida, Stefany P. Costa, Jonas Botelho, William F. Laurance

**Affiliations:** ^a^Centre for Tropical Environmental and Sustainability Science, College of Science and Engineering, James Cook University, Cairns, QLD 4878, Australia; ^b^Department for the Ecology of Animal Societies, Max Planck Institute for Animal Behavior, Konstanz 78467, Germany; ^c^IMAZON-Amazon Institute of People and the Environment, Belém, PA 66055-200, Brazil; ^d^Institute of Environmental Planning, Faculty of Architecture and Landscape Sciences, Leibniz University, Hannover 30419, Germany; ^e^Ecosystem Management Group, Department of Environmental Systems Science, ETH Zurich 8092, Switzerland

**Keywords:** infrastructure, impact assessment, conservation, Amazon, development

## Abstract

Roads act as conduits for human incursions and hence underlie many of humanity’s impacts on nature, including deforestation, wildfires, and natural-resource overexploitation. Unfortunately, existing roadmaps often drastically underestimate the true extent of road networks and future predictions of road-related impacts rely on incomplete and outdated data, undermining development planning and conservation decision-making. Here, we develop a multivariate “road expansion risk” index to identify areas prone to road building and therefore vulnerable to road-related environmental impacts. Using a massive road dataset—137 million 1-ha raster cells drawn from three different sources arrayed across the Amazon and Congo basins and insular Asia-Pacific region—we predict road-prone locations via a statistical model that integrates a range of biophysical, socioeconomic, and administrative data. This highly integrative, large-scale approach allowed us to identify areas likely to experience future road building and regions that may contain unmapped roads. Importantly, our road expansion risk index is a strong predictor of forest loss and degradation and can hence identify future road building and deforestation hotspots, even for the many tropical forest locales with grossly deficient road data.

## Significance

This study identifies consistent biophysical and socioeconomic predictors of road building across the world’s major tropical rainforest regions. With this information, we devise a multivariate road expansion risk index that effectively identifies areas likely to bear unmapped roads or that are susceptible to future road building. Beyond predicting the risk of roads, the index can also predict the probability of deforestation, even for locales without road data. By disentangling the various factors influencing the contemporary explosion of tropical roads and associated deforestation, we present an important tool for use in various conservation planning and assessment practices, including protected area design and management and threatened species risk assessments, and for forecasting hotspots of human incursion into tropical forest regions.

In forested regions, new roads can trigger many destructive impacts on nature, including deforestation (1, 2), wildfires (3), and overexploitation of fauna and flora (4), among others (5). Unfortunately, the management of roads is challenging because existing datasets dramatically underestimate road networks in many nations (6–9). Such road networks, furthermore, are constantly expanding (10–13), undermining efforts to conserve ecosystems and biodiversity (9). Hence, understanding where new road building and road-related deforestation are likely to occur—particularly in data-poor regions—would be of substantial value for conservation planning and decision-making. For these reasons, we devised a “road expansion risk” model that predicts where new roads and deforestation are likely to occur across the world’s major tropical forest regions, while also accounting for regionally varying drivers of development.

In the tropics, road building is proceeding apace (11, 14–16) and often immediately precedes deforestation, especially in remote frontier areas (9, 17). However, not all areas are suitable for roads. Road building is strongly influenced by local factors such as topographic slope (18) and soil chemical and physical properties (19). Climatic variables such as rainfall, socioeconomic factors such as population density and economic growth, and administrative variables such as governance region also affect the costs of road construction and maintenance (20–23). Given that the ultimate causes of deforestation, such as mining, logging, and agriculture, are largely confined to areas suitable for road building (24–26), identifying the factors that allow road proliferation is a key priority.

We propose a road expansion risk index as a powerful tool for predicting and safeguarding future hotspots of deforestation. Here, we 1) use road data from the Brazilian Amazon, Congo Basin, and tropical Asia-Pacific region to identify correlates of landscape suitability for road building and then compare these patterns among the geographic regions; 2) develop a road expansion risk index that is robust to regional variation in drivers of development and predicts landscape suitability for road building; and 3) demonstrate that our index can effectively predict patterns of forest destruction even with a complete absence of actual road data.

## Results

### Correlates of Road Building.

We obtained road locations from leading published road datasets (8, 9, 11) and used these to create road presence–absence maps at 1-ha resolution. These maps covered the Congo Basin, Brazilian Amazon, and insular Asia-Pacific region, respectively—all regions with exceptional environmental and socioecological values that are under threat from increased anthropogenic pressures (25, 27, 28).

**Fig. 1.**
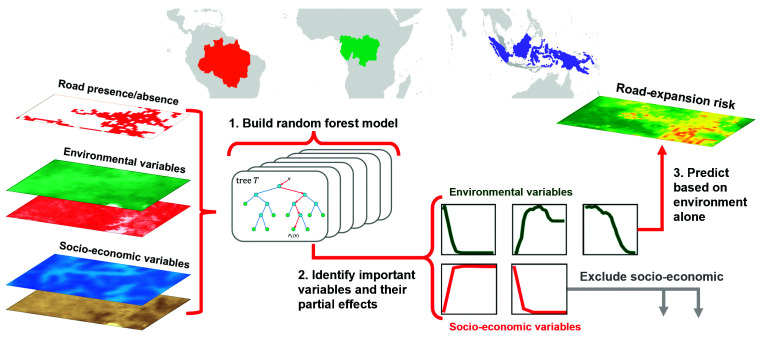
Conceptual layout of study region and methodology for road expansion risk modeling.

We then created a model training sample of 137 million 1-ha raster cells, of which 47 million were road presences, and extracted information for 44 different biophysical, socioeconomic, and administrative variables expected to influence road building (*Materials and Methods*). While the aim was to identify biophysical conditions that enable road construction, we included socioeconomic and administrative variables to account for their influence on road construction rates and create a more realistic assessment of the impact of underlying environmental constraints. For example, we included national and subnational administrative regions to account for different rates of road construction among governance regions. Alternatively, variables such as human population density may have a more complex relationship with road construction; roads can initially act as a driver by allowing access for land-colonization (either initial settlement or invasion and land-grabbing in existing small settlements) (1), but as the population density increases it itself becomes a driver of road construction as more infrastructure is required to support the population (20). We used our extensive dataset to build region-specific and pantropical random-forest models of road building suitability and developed a “road expansion risk index” based on biophysical suitability alone (Fig. 1). Because drivers of both road building and forest loss and degradation vary within and among countries (25, 29), our biophysical road expansion risk index is robust to variations in such socioeconomic and administrative factors.

**Fig. 2.**
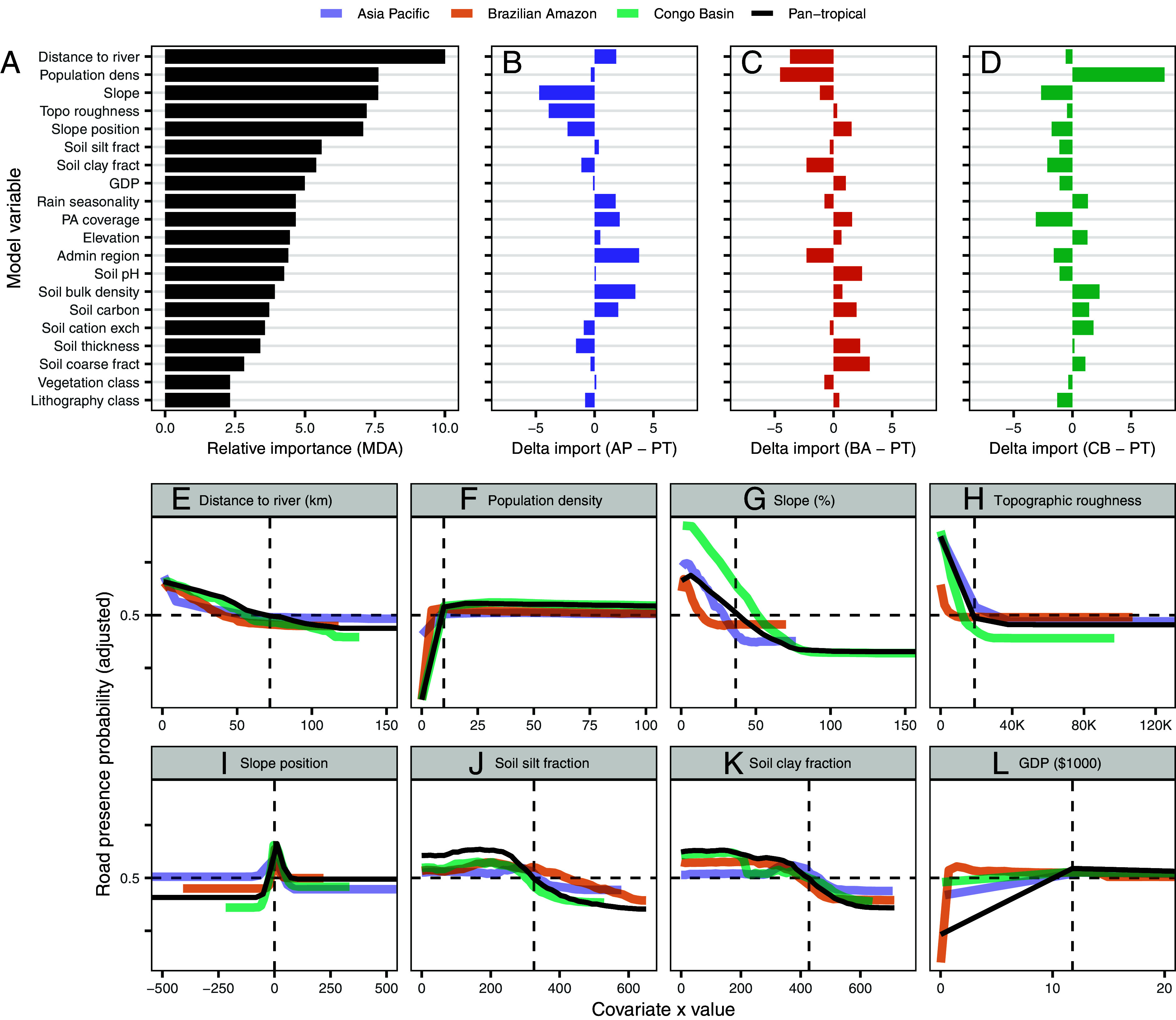
Spatial predictors of road presence when modeling using a pantropical dataset or modeling continental regions separately. (*A*) Relative importance (mean decrease in accuracy when permuting) for model variables in the pantropical model. (*B*–*D*) Difference in relative importance for model variables between the pantropical model and (*B*) the Asia-Pacific model, (*C*) Brazilian Amazon model, and (*D*) Congo basin model. (*E*–*L*) Partial differential plots for the eight most important model variables, for the pantropical model and all three region-specific models. Horizontal dashed lines indicate the 0.5 threshold value when roads are more likely to be present than absent. Vertical dashed lines indicate the first value for the model term at which the 0.5 threshold is crossed (except for slope position in which it is located at 0).

Our final model included the 20 most influential correlates of road presence out of our initial list of 44 potential covariates (*Materials and Methods*). The most influential covariates in the pantropical model were distance to river, population density, and topographic features such as slope and topographic roughness (Fig. 2*A*). Soil characteristics such as silt and clay fraction were also highly important, as was rainfall seasonality (measured as the coefficient of variation among months). Vegetation class alone—of which wetlands were one class—did not have a strong influence on model accuracy, but this may be due to other correlated variables having greater explanatory power (i.e., soil and rainfall characteristics) or due to inconsistent patterns of vegetation management across the study region [i.e., drainage of peat swamps for agriculture is common in the Asia-Pacific region (30) but much less so in the Congo basin at present (31)].

The importance of various predictor variables was generally similar among all models (Fig. 2 *B*–*D* and *SI Appendix*, Fig. S1), apart from population density, which was substantially more influential in the Congo Basin, and topographic variables, which were less influential in the Asia-Pacific region. The Congo Basin has lower rates of commodity-agriculture-driven deforestation than the Brazilian Amazon and Asia-Pacific regions (25, 29), a land-use system that is often associated with extensive road network expansion in the absence of high population densities (9). While selective logging promotes significant road building in the Congo basin irrespective of population density, these road networks are typically sparser than those associated with commodity agriculture (i.e., oil palm plantations or soy cropping common in the Asia-Pacific region and Brazilian Amazon, respectively) and more ephemeral (11, 32). Conversely, the most notable driver of forest loss in the Congo Basin is small-scale and subsistence agriculture (25, 29, 33) linked closely with road infrastructure and human populations (33).

Partial relationships between covariates and road presence were also largely consistent across regions and in the pantropical model (Fig. 2 *E*–*L* and *SI Appendix*, Fig. S2 *A*–*L*). Relationships followed expected trends, with roads more likely to occur in dry, flat areas, in regions characterized by inorganic soils, close to rivers (which provide alternative access points for human incursion), or in populated areas. Given that vegetation type had little influence in the model and the key correlates of road building are primarily topographic and soil conditions, we suggest that our findings are largely valid in regions outside of our study region, including other areas of tropical moist and dry forests. However, vegetation types other than moist forests were underrepresented in the study region; hence, further work is necessary to confirm the transferability of the models. Notably, the rank-encoded categorical variables we used did not have linear or monotonic relationships with road presence (*SI Appendix*, Fig. S2). This is particularly relevant in the case of subnational administrative regions as it suggests that, while influential, governance or other administrative-level factors (such as environmental policy) did not have substantial power in determining road building when contrasted against landscape factors and population density—however, this has not been assessed here in detail. Encouragingly, however, protected-area coverage reduced the probability of road building in all models, supporting conclusions from Engert et al. (9) that protected areas often reduce environmental and social impacts by minimizing road incursions.

Importantly, our pantropical model had reasonably high predictive performance, with a mean AUC = 0.802. Given the inherent complexity of predicting locations of roads (i.e., due to connectivity and topological attributes; (8)), we consider this to be a robust model. The performance of the pantropical model also differed among regions, with a mean AUC = 0.746 for the Asia-Pacific region, AUC = 0.850 for the Brazilian Amazon, and AUC = 0.809 for the Congo Basin (*SI Appendix*, Figs. S3–S5). While the region-specific model for the Asia-Pacific performed better than the pantropical model (AUC = 0.850), we opted to use only the pantropical model for further analyses as it performed better than the region-specific models for the Brazilian Amazon (AUC = 0.847) and the Congo Basin (AUC = 0.792), and to overcome any regional biases that may be present in training data or region-specific land-use practices. For example, topographic variables were less influential in the Asia-Pacific region, where there is substantial deforestation in sloping terrain and at higher elevations (34).

While some roads, such as those within logging concessions, are often abandoned after construction (11), we did not consider road persistence in our model as we focused on only identifying factors affecting road building. However, it is important to note that road persistence and long-term ecological impacts are also influenced by a range of socioeconomic and environmental factors (11, 32).

### Road Expansion Risk.

**Fig. 3.**
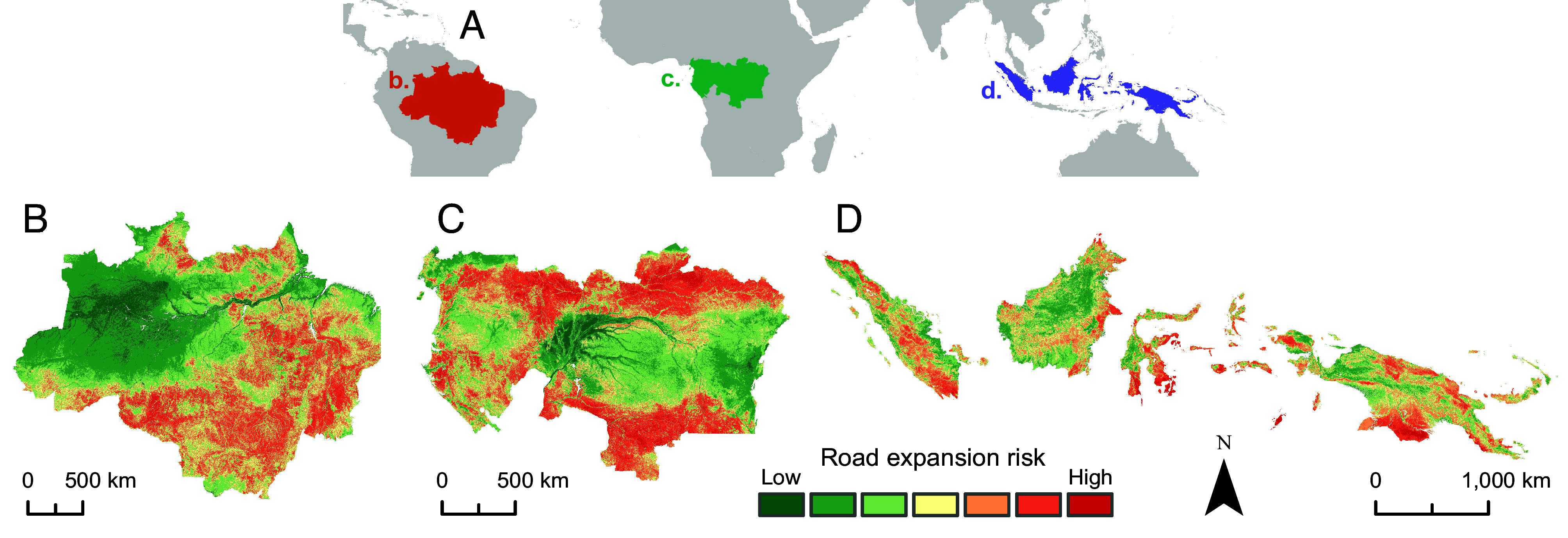
Predicted road expansion risk (adjusted road suitability) as calculated using the pantropical model. (*A*) study region extent indicating delineated continental regions: Brazilian Amazon (orange), Congo basin (green), and Asia-Pacific (purple). (*B*–*D*) model predictions for the aforementioned continental regions.

We developed a spatial index of road expansion risk using the modeled partial relationships between environmental variables and road presence (Fig. 3). This layer categorizes the suitability of land for road building based on various biophysical features while accounting for the influence of socioeconomic and administrative factors, such as increased road construction on steep slopes and in densely populated areas. Our models suggest that much of the Amazon and Congo regions have high road expansion risk, with their extensive wetlands being less susceptible. Some areas, such as central Sumatra, can experience substantial roading even when appearing to be low-risk, for example, due to high population densities or local land-use practices—such as peat-swamp drainage (30)—that predispose even nonoptimal locales to roading and related environmental impacts. Hence, while our index identifies areas with biophysical conditions that allow or constrain road building, socioeconomic information (i.e., population density and local-development drivers) is also important.

We identified several regions—such as the Guyana Shield in Amazonia, Congo Basin rainforests, and much of New Guinea—with high road expansion risk scores but have not yet experienced substantial land colonization and conversion (35). These areas are of particular conservation concern as they are often the subjects of proposed future developments, including highways, development corridors, and major oil palm and wood-pulp projects and logging concession (11, 27, 31, 36, 37). For example, large tracts of the Congo basin peat-swamp forests have seen little road building due to accessibility constraints and logging moratoriums but are likely to come under threat from logging and oil palm concessions with increases in global resource demand (31). Similarly, areas of eastern Kalimantan, Indonesia, have only low to moderate road development but are vulnerable to future road expansion precipitated by the development of Pan-Borneo highways and the new Indonesian capital city of Nusantara in eastern Borneo (38, 39). Our road expansion risk map can be used to identify and help mitigate environmental degradation in such areas prone to future roading and deforestation.

In the Brazilian Amazon, many vulnerable areas are located within protected areas or Indigenous territories. In such locales, strong governance and legal support are essential to help protect forests and their biodiversity (40, 41). Our modeled road expansion risk layer can be used to identify areas at risk of future road incursions so that policy actions and enforcement activities can be appropriately focused to maximize positive outcomes.

### Road Expansion Risk Predicts Deforestation.

**Fig. 4.**
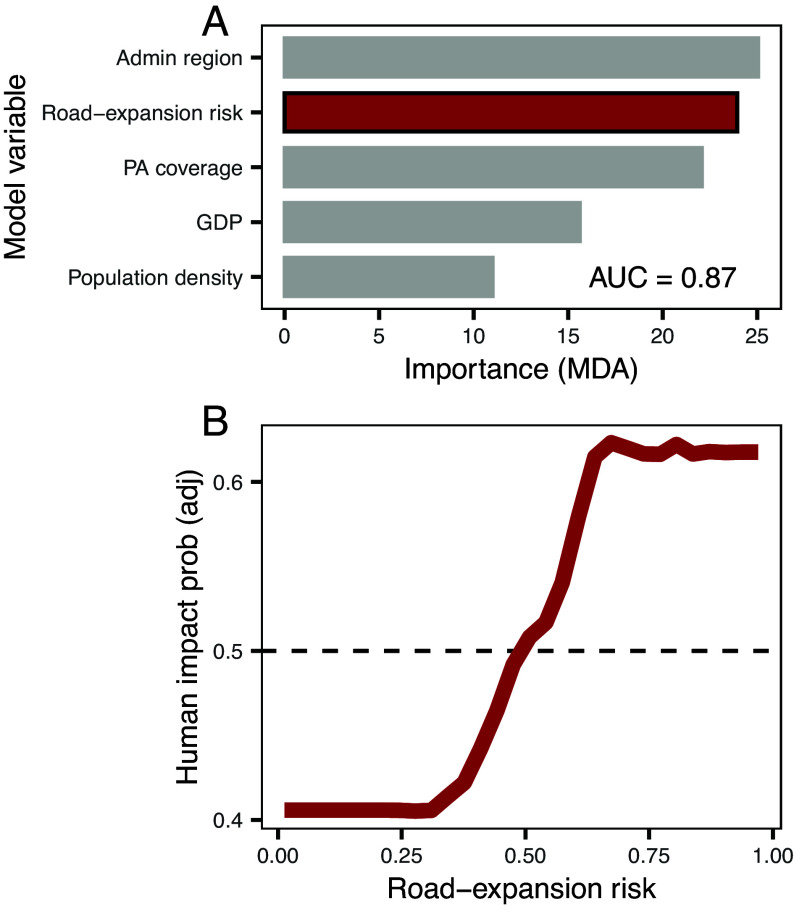
Road expansion risk (probability of road presence) as a spatial predictor of human impacts. (*A*) Importance of model variables (mean decrease in model accuracy when variable is permuted) for the five variables retained in the final model. (*B*) Partial differential plot showing the relationship between road expansion risk and probability of human impact presence. Human impacts were forest loss and forest degradation identified using the Vancutsem et al. (45) dataset.

Roads act as key proximate drivers of tropical deforestation by promoting activities such as commodity agriculture, logging, and mining (24, 26). Hence, our index of road expansion risk index should be a reliable predictor of forest destruction. Using a simplified model with only five variables—one of which was road expansion risk—we were able to predict forest loss and degradation with high performance (AUC = 0.870; Fig. 4). The other four variables were administrative region, population density, GDP, and protected areas (*SI Appendix*, Fig. S6). Our model’s predictive performance is comparable to models with many more variables (including high-resolution road locational data, and landscape fragmentation and deforestation metrics; i.e. (9, 42). Despite having little influence on road building, administrative region was the strongest predictor of forest loss and degradation in our simplified model, highlighting the role of governance in mediating the effects of roads and development on rates of environmental destruction (43, 44).

Our road expansion risk model can be used for many conservation-related activities. For example, it can be used to evaluate the performance of protected areas (9, 46, 47) and identify regions likely experiencing de facto protection due to their unsuitability for development. Similarly, the road expansion risk index can be used to identify “roadless” and “wilderness” areas that may currently host unmapped roads (7, 9) or be threatened by future road expansion and large-scale land conversion (48, 49). The road expansion risk layer can also identify areas at risk from a range of other anthropogenic disturbances, including illegal logging and mining (26).

## Discussion

Using a massive pantropical dataset, we identified robust correlates of road building in forested regions. After accounting for the local relevant socioeconomic and administrative context, road building was influenced most by rainfall patterns, soil conditions, topographic variables, and proximity to rivers. With this information, we created a road expansion risk index that allows us to reliably predict forest loss and degradation locations, thereby overcoming the badly incomplete road datasets typical of many tropical nations (5, 9).

Roads are strong and consistent spatial predictors of deforestation (2, 9, 50). As such, they are vital for predicting future deforestation frontiers and hotspots. However, deforestation models are hampered by static—often seriously outdated—road data (e.g., 51), with millions of kilometers of additional roads being constructed annually (10, 13). Crucially, our index of road expansion risk can both identify areas likely to be under threat from deforestation without requiring road data, and proactively identify areas in which future road building, and hence, deforestation hotspots, are most likely to arise (52–54). This advancement is particularly relevant given the explosive growth of road networks and other infrastructure across the global tropics, including scores of massive development corridors across Africa (55, 56), South America (57), and the Asia-Pacific region (36, 58).

Because roads are almost universal vectors of human encroachment, our road expansion risk index has many uses outside of land-use-change modeling. For example, roads play a key role in spreading human pathogens (59–61), and our index can be used to identify high-risk areas for zoonotic-pathogen spillover into human populations (62, 63). Similarly, our index can be used to identify likely invasion routes for exotic weeds and feral animals, which commonly proliferate along roads and in human-disturbed ecosystems (64, 65). Finally, frontier roads can be destabilizing socially. They can bring benefits such as improved access to markets and social-support systems (66) but also create major risks—such as when land invaders or land speculators displace forest peoples by violently dispossessing them of their land and natural resources (67–69). By projecting where new roads are most likely to proliferate, our road index can be used to identify areas in which new roads designed to improve socioeconomic conditions for rural communities may also lead to explosive secondary-road building and land invasions.

Road development is determined by human activities and decisions, with resource extraction and transport being key drivers (9, 70). Hence, our metric based on suitability for road building may be influenced by suitability for practices such as agricultural production and forestry, as well as proximity to ports and other transport hubs. We have aimed to account for such factors by utilizing a complex subsampling strategy and development of a pantropical model so that no specific land-uses dominate, as well as inclusion of various socioeconomic and administrative variables in model training. However, it is not possible to account for the multiplicity of historical legacies and political and economic interests that shape road building trajectories (8, 9, 11, 45), which may lead to local diversions from our predictive model. Overall, we do not consider these aspects to be a substantial limitation, as areas identified as having high road expansion risk are still expected to be under elevated threat even if road development is driven by factors that remain unaccounted for in our model.

## Materials and Methods

### Study Region.

Our study region encompassed tropical wet forest regions for which high-quality, recent road data were available. This included the rainforests of the Congo Basin (study region borders defined by subnational administrative regions predominantly covered by moist forests) covered by Kleinschroth et al. (11), the Brazilian Amazon covered by Botelho Jr. et al. (8), and the insular Asia-Pacific region covered by Engert et al. (9). We focused on tropical wet forests as this was the ecosystem type predominantly covered by the above datasets, and because these regions were also covered by the Vancutsem et al. (45) forest disturbance dataset. While relying on a single forest cover or forest loss dataset has limitations, the Vancutsem et al. (45) dataset appeared to be the most robust single forest loss and degradation dataset for our study region at the time of assessment (based on visual comparison). The regions covered in this study are also characterized by exceptionally high biodiversity, carbon, and socioecological values (28, 71), and high current or future rates of economic development and commodity-driven land conversion (25, 27).

### Road Data.

We obtained the most recent, highest quality road data available for each of the continental regions covered by the study, including data produced by Kleinschroth et al. (11), Botelho Jr. et al. (8), and Engert et al. (9). Kleinschroth et al. (11) and Engert et al. (9) road datasets were manually digitized using satellite imagery, while Botelho Jr. et al. (8) was created using automatic road detection from satellite imagery. As these three road datasets were primarily focused on digitizing roads outside of populated areas, we supplemented the datasets with the most recent Open Street Map data (circa 2023). We selected all roads in the Open Street Map data that did not intersect roads in the aforementioned datasets and appended them to the relevant road dataset using Select Layer by Location and Append tools in ArcMap 10.8. While the three road datasets are from different sources and are generated using two different methodologies (automatic detection from satellite imagery and manual digitization from satellite imagery), extensive visual inspection determined that the datasets were comparable in terms of accuracy and precision. While manual road digitization may occasionally have lower spatial precision, this is accounted for by converting road vector data into presence/absence rasters at 1-ha resolution. Further, differences in accuracy or completeness of spatial datasets may be accounted for by including national and subnational administrative regions in the model. While temporal data on road expansion rates would be highly suited for the analyses conducted, such data do not currently exist with high accuracy or high spatiotemporal coverage.

### Model Variables.

Our response variables for the two models were 1) road presence or absence and 2) presence or absence of human impacts. Road presence or absence was quantified by converting the aforementioned road datasets to a raster layer at 1-ha resolution (presence) using the Feature to Raster tool in ArcMap 10.8 and mosaicking onto a map of the study region extent (areas not covered by the presence layer considered absences). Human impact presence or absence was quantified by first reclassifying the Vancutsem et al. (35) Transition Map—Subtypes data as either “human impact” or not, following Engert et al. (45) at 25 m raster cell resolution. We aggregated this layer to a 1-ha raster resolution by calculating the proportion of each 1-ha cell that had some human impact. This was then converted to a binary presence absence layer using a cut-off threshold of 10% impacted to remove cells where natural disturbances may have been misclassified as human impacts following Engert et al. (45). The 10% threshold value was chosen based on extensive visual inspection of the forest cover data (45).

We created an extensive list of potential correlates of both road construction suitability and road-related deforestation based on published literature and hypothesized relevance (*SI Appendix*, Table S1). While the aim was to identify biophysical conditions that enable road construction, we included socioeconomic and administrative variables in the model for multiple reasons. We included national and subnational administrative regions, for example, to account for differences in road construction rates between governance regions. Alternatively, variables such as population density may have a more complex relationship with road construction where roads initially act as a driver by allowing access for colonization of land, but as the population density increases it itself becomes a driver of road construction as more infrastructure is required to support the population. We therefore included such variables to account for their influence on road construction rates relative to underlying environmental constraints. While some of these variables may not typically be listed as “socioeconomic” factors, we have included them within this broad category for simplicity in grouping model terms and because they influence social and economic activity in some way. Additionally, previous work has shown that many correlates have neighborhood effects (9, 42); hence for several covariates, we calculated mean values over varying focal window sizes (values averaged over all cells within a specified distance). For example, population centers (areas with high population density) can increase the demand for resources that drive increased rates of development and deforestation in neighboring areas. Our initial list included 44 potential correlates (*SI Appendix*, Table S1). All spatial data for model variables were obtained from published or authoritative sources.

As the model sampling and iteration structure we employed is unsuitable for handling categorical variables, we converted these from categorical to numeric terms using rank encoding. Rank encoding allows categorical variables to be considered as numeric variables, while reducing the amount of information leakage when compared to target-encoding (72). Using the Focal Statistics tool in Arcmap 10.8, we calculated the mean road presence and human impact presence for each class of each categorical variable (country, subnational administrative region, preclearing vegetation class, protected area governance, protected area IUCN class, lithography class) across the study region and ranked the classes using these values. For protected area governance and protected area IUCN class, we created a basic rank encoded value which did not consider differences between countries (a single rank value for each class irrespective of country), and a second rank encoded value that did consider differences between countries (a rank value for each combination of protected area class and country, i.e. private protected areas in Brazil having a different rank than private protected areas in Malaysia).

### Sampling.

Our study region included ~866 million 1-ha raster cells, of which ~53 million cells contained roads. Therefore, to reduce computational burden and imbalance in the response variable, we created a model training sample of ~137 million 1-ha raster cells, including ~47 million cells containing roads. Our training sample included 15 million observations for the Congo Basin (5 million with roads), 32 million for the Asia-Pacific region (12 million with roads), and 90 million for the Brazilian Amazon (30 million with roads). To ensure our model training sample was representative of the entire study region, we compared the value distributions of model variables between the model training and total datasets. Visual inspection revealed that the distribution of values in the model training sample was near-identical to those in the total dataset (*SI Appendix*, Figs. S9–S11). We opted not to conduct a statistical test to compare the distributions as the number of observations in each class would render even trivially small differences significant. To further reduce computational burden and model training time, we divided each region into subsamples of ~1.5 million points, with 500,000 road presences and 1 million road absences (10 subsamples for the Congo basin, 20 for the Asia-Pacific region, and 60 for the Brazilian Amazon).

### Model Routine.

To identify spatial determinants of road construction, we created random forest classification models with road presence or absence as the response variable. Random forest models are built by combining multiple independent classification trees, and as such are robust to complex nonlinear and nonmonotonic relationships and collinearity (73). We considered our modeling routine to be similar to a presence-background formulation, for which previous work has demonstrated the high performance of down-sampled random forest models (74), for two key reasons. Namely, 1) road locations are temporally variable as roads may be abandoned following construction and road building and abandonment are dynamic ongoing processes; and 2) road detection is imperfect both when using manual digitization and when using AI road detection algorithms, due to mapper error, old or low-resolution satellite imagery, roads obscured by tree canopy cover, or imperfect detection algorithms. We additionally chose to use random forest models because trees can be trained independently on separate data samples and combined into a final model, which allowed us to develop a complex data subsampling method (*SI Appendix*, Fig. S7) to improve computational efficiency and minimize overfitting.

To ensure that each continental region was equally represented in model training despite imbalances in our model training sample (i.e., more training data from the Brazilian Amazon than the Congo Basin), we built the random forest model by training on one subsample (1.5 million observations) from each region in each iteration (4.5 million observations total per iteration). As road density is highly variable between regions, there were uneven numbers of subsamples (60 in the Brazilian Amazon, 20 in the Asia-Pacific region, and 10 in the Congo basin). To minimize the impact of the differences in road density, each subsample for the Brazilian Amazon was used once, each from the Asia-Pacific used twice, and each from the Congo basin used six times in total. The random forest was therefore built by iterating through 60 iterations each containing 45 trees, for a total of 2,700 trees. To further reduce computational burden and minimize risk of overfitting, individual trees in each iteration were trained on a sample containing 100,000 presences and 100,000 absences, and sampling was conducted with replacement so that each individual tree was trained on a different portion of the subsample (*SI Appendix*, Fig. S7). Random forest models were fit using the randomForest package (75) in R (76).

Spatially structured data are subject to spatial autocorrelation, the presence of which violates the assumption of independence and can inflate Type I error rates and lead to selection of unimportant explanatory variables and poorly estimated model parameters (77, 78). Hence, to account for spatial autocorrelation in our model, we developed a spatial autoregressive (SAR) term as inclusion of such a term in model fitting is less likely to bias model-parameter estimates than other methods (79). To confirm that spatial autocorrelation had been adequately accounted for in the final model, we calculated Moran’s I on model residuals using the moranfast (80) package.

We therefore developed the final road suitability model through three model generations (*SI Appendix*, Fig. S8). In the first generation, we ran the random forest model with all 44 potential correlates and determined the variable importance. We then removed all variables with an importance (mean decrease in permutation accuracy) of 0. Additionally, for variables that were measured in different ways (protected area governance vs protected area IUCN category) or at different spatial scales (population density at different neighborhood size) we kept only the version with the highest importance value, leaving 20 correlates (*SI Appendix*, Fig. S12). We then reran the model with this reduced list of covariates to create the second-generation model. This second-generation model was then used to predict road presence across our entire dataset (866 million ha) and create a spatial autoregressive (SAR) term from the model residuals following Crase et al. (79). Finally, we reran the model again, including this SAR term to create the third-generation model. This third-generation model was the final model for which variable importance, partial plots, model performance, and model predictions were calculated (*SI Appendix*, Fig. S8). Model performance and model predictions were calculated holding the SAR term at a value of 0 to negate its influence on both performance and prediction. Model performance was calculated through 100 iterations of 100,000 randomly sampled observations for each continental region (30 million total observations) from the full dataset at the original prevalence ratio rather than the training sample in order to avoid overestimating predicted probability of road presence or increasing the false positive rate. We then averaged the performance metrics across these iterations to give mean AUC (Area Under the receiver operating characteristic Curve) values. Moran’s I was calculated using the same 100 iterations of 100,000 randomly sampled observations. Moran’s I values were low (mean = 0.07, sd = 0.01), and hence, we considered that spatial autocorrelation was adequately handled.

### Sensitivity Analyses.

To ensure that our data sampling strategy did not adversely affect model performance, we conducted sensitivity tests. By downsampling road presences in order to balance the two classes in model fitting, we artificially increased the road prevalence ratio relative to the full dataset, which may lead to overestimated road presence probabilities and influence the relative importance of model variables. We therefore constructed alternative models using the aforementioned modeling framework but with two key changes 1) a model trained on data samples using the original road prevalence ratio, and 2) a model trained on data samples using the original road prevalence ratio and with class weights applied to handle the class imbalance.

After constructing models using the initial model framework (referred to as the “balanced sample” model) and the two alternative models on a representative subset of the data containing 44 million observations, we compared model predictive performance and variable importance. First-generation model variable importance values—measured as the mean absolute SHapley Additive exPlanations (SHAP) values—were largely consistent across the three modeling frameworks, and variables retained for the second-generation model were largely the same (*SI Appendix*, Fig. S13), and there was a strong correlation between SHAP values for model variables among the models (S14). This suggests that the data sampling structure employed did not have a substantial influence on relative variable importance.

After creating spatial autoregressive terms for each of the three alternative modeling frameworks and constructing the third-generation models, we compared the predictive performance and explanatory power. Model predictive performance was calculated by taking a sample of 100,000 observations at the same road prevalence ratio as the full dataset (*SI Appendix*, Fig. S7) and calculating the AUC values. This step was iterated 25 times for each model to give mean AUC values based on 2.5 million observations. AUC values were consistently and significantly highest for the balanced sample model (mean = 0.848, sd = 0.002; *SI Appendix*, Fig. S15*A*). The model using the original road prevalence ratio (mean = 0.834, sd = 0.003), and the model using class weights (mean = 0.834, sd = 0.002) had lower predictive performances. Finally, model variables had, on average, higher explanatory power (measured as the mean absolute SHAP values) in the balanced sample model than in either of the alternative models (*SI Appendix*, Fig. S15*B*). Hence, we determined that the balanced sample method we employed was the most suited to our aims.

Finally, as the performance of the sensitivity test models was high (mean AUC = 0.848 for the balanced sample model), we compared models in which variables were selected 1) based on the highest mean decrease in permutation accuracy (MDA) and 2) based on the highest mean absolute SHAP values. When selecting by mean absolute SHAP values, there were slight differences in which variables were retained in the final model (*SI Appendix*, Fig. S16)—such as mean rainfall in a 1 km radius in the SHAP model versus rainfall seasonality in a 5 km radius in the MDA model, and population density in a 50 km radius in the SHAP model versus population density in a 5 km radius in the MDA model—however, the majority of variables retained were the same. Further, the performance of the third-generation SHAP model was slightly lower than the third-generation MDA model (mean AUC = 0.778 versus mean AUC = 0.802, respectively); hence, we opted to report only the MDA model. The decreased performance of the SHAP model may be due to retention of variables with nonuniform relationships with the response variable (road presence) across the study region, for example, soil pH had a high mean absolute SHAP value (*SI Appendix*, Fig. S16) but has a positive relationship to road presence in the Asia Pacific region and a negative relationship in the Congo and Amazon Basin regions (*SI Appendix*, Fig. S2*C*).

### Region-Specific Models.

After the first model generation and removal of redundant covariates, we used the new list of 20 model covariates to create continental-region-specific models. These models each followed the same second and third generations as the pantropical model (run second generation and predict to create the SAR term, run third generation including the SAR term). We then used these three continental models (for the Brazilian Amazon, Congo Basin, and Asia-Pacific) to compare 1) the importance of model covariates across regions, 2) the partial effect of covariates on road building across regions, and 3) the predictions and performance of the continental models against the region-wide model.

### Road Expansion Risk.

After developing the third-generation model of road suitability, we developed a road expansion risk layer based on the environmental variables alone. To do this, we predicted the probability of road presence while holding all socioeconomic and administrative variables (population density, administrative region, etc.) at either their mean value or zero. This method allowed us to preserve the partial relationship between environmental variables and road presence accounting for the effect of socioeconomic and administrative variables, but ignoring the influence of the socioeconomic and administrative variables in the prediction surface. We then used the dismo package (81) to identify the value for this prediction surface for which specificity and sensitivity were maximized and rescaled the prediction surface so that this value became 0.5, the minimum value became 0, and the maximum became 1. This final adjusted probability layer was termed the “road expansion risk” layer.

### Road Expansion Risk Drives Deforestation.

To examine the effect of road construction suitability as a correlate of human impacts, we developed a similar random forest model routine as used to identify drivers of road presence. We followed the same data sampling and model fitting structure as outlined for road suitability; however, for this model, we used human impact presence or absence (as classified in (29) as the response variable. Model variables included the road expansion risk layer as well as the socioeconomic and administrative variables related to population density, GDP, governance regions, and protected area coverage.

## Supplementary Material

Appendix 01 (PDF)

Code S01 (R)

## Data Availability

R scripts detailing data sampling and modeling methods, and all data used to create manuscript figures, are available as Supplementary Data files. Brazilian Amazon road data and derived datasets (i.e., road presence) are under copyright in the PrevisIA platform (https://previsia.org.br/) and cannot be stored in a public repository, but will be made available to researchers on request by Dr. Carlos Souza Jr. (souzajr@imazon.org.br). All other spatial datasets are freely available from their respective data sources (*SI Appendix*, Table S1).
