# Supplementary material for: Road expansion risk predicts future hotspots of tropical deforestation
Source: Proc Natl Acad Sci U S A. 2025 Dec 22;122(52):e2502426122. doi: 10.1073/pnas.2502426122 (PMC12771565; doi:10.1073/pnas.2502426122)
Supplement: Supplementary file 1 — Appendix 01 (PDF) [file pnas.2502426122.sapp.pdf]

Supplementary Materials

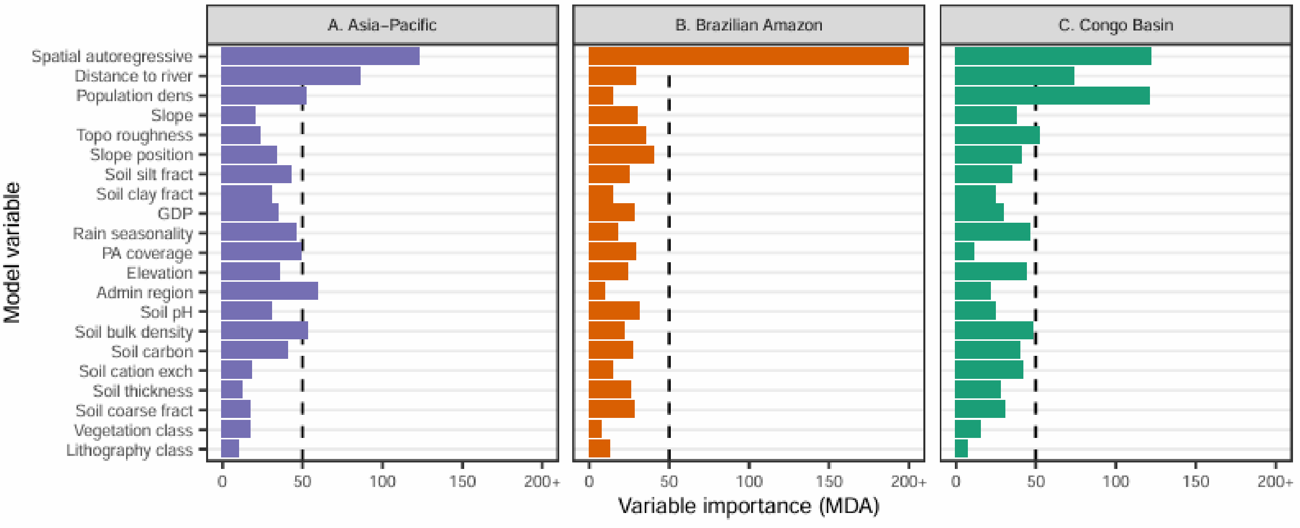

Fig. S1. Actual values of variable importance (mean decrease in model accuracy when permuted) for model variables in each region-specific model. The spatial autoregressive (SAR) term had substantially higher importance in the Brazilian Amazon than in either Asia-Pacific region or Congo Basin.

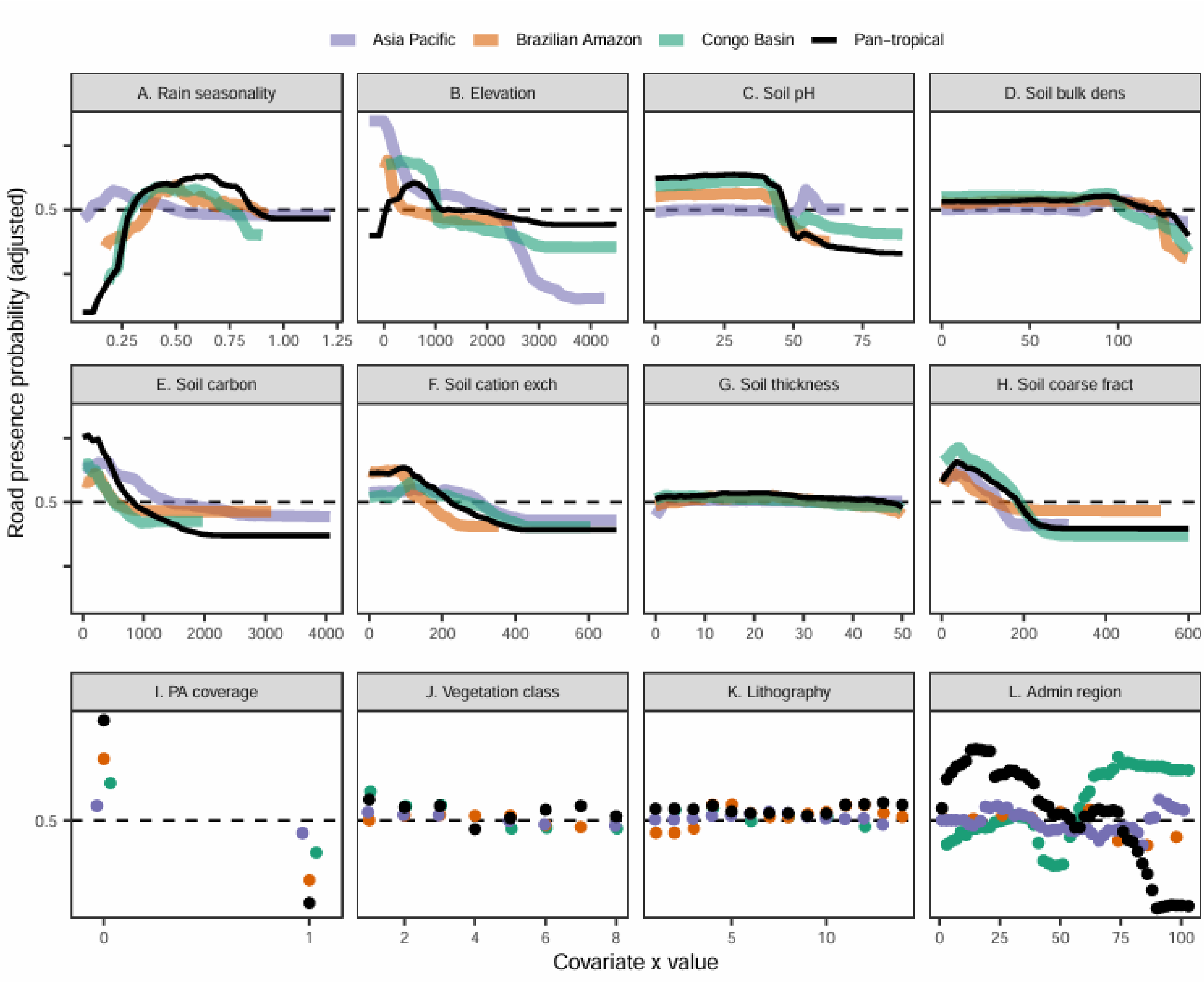

Fig. S2. Partial differential plots for the 12 variables not shown in the main text (less important as determined using mean decrease in accuracy when permuted).

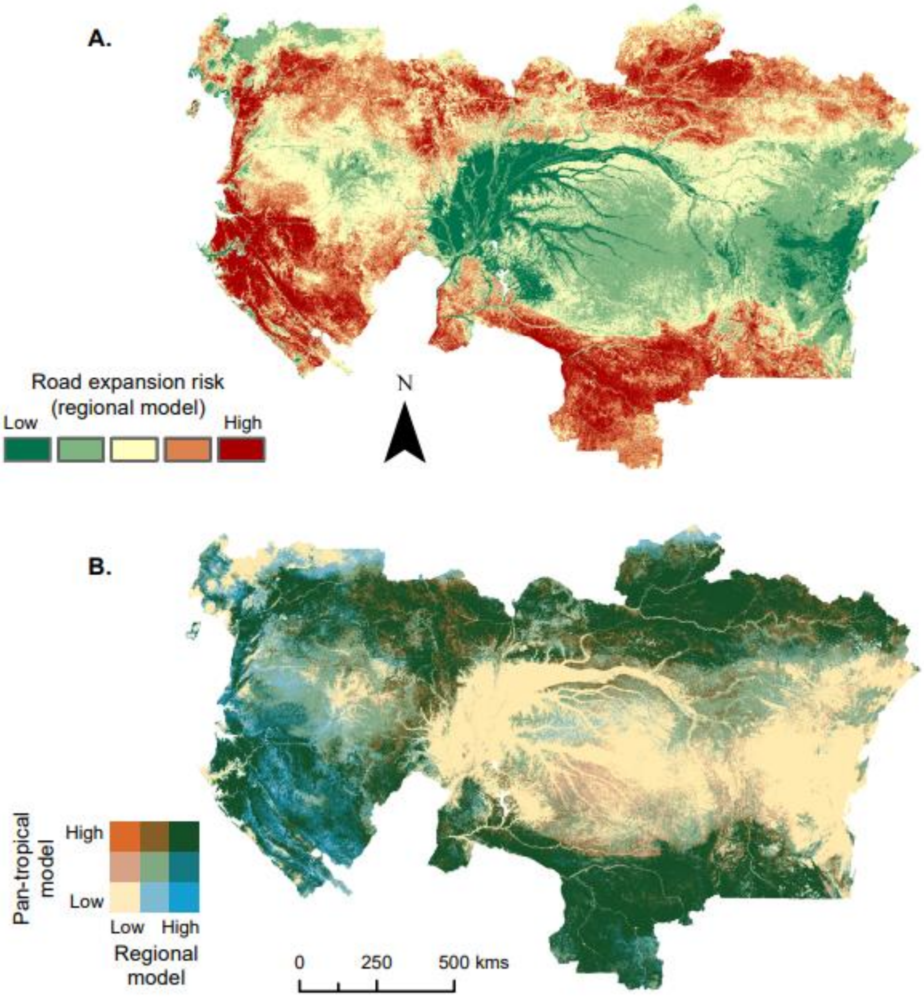

Fig. S3. Road-expansion risk from the region-specific model for the Congo Basin and comparison to predictions from the pantropical model. For the comparison plot, low-medium-high thresholds are as follows: low = 0 – 0.45, medium = 0.45 – 0.55, high = 0.55 – 1.

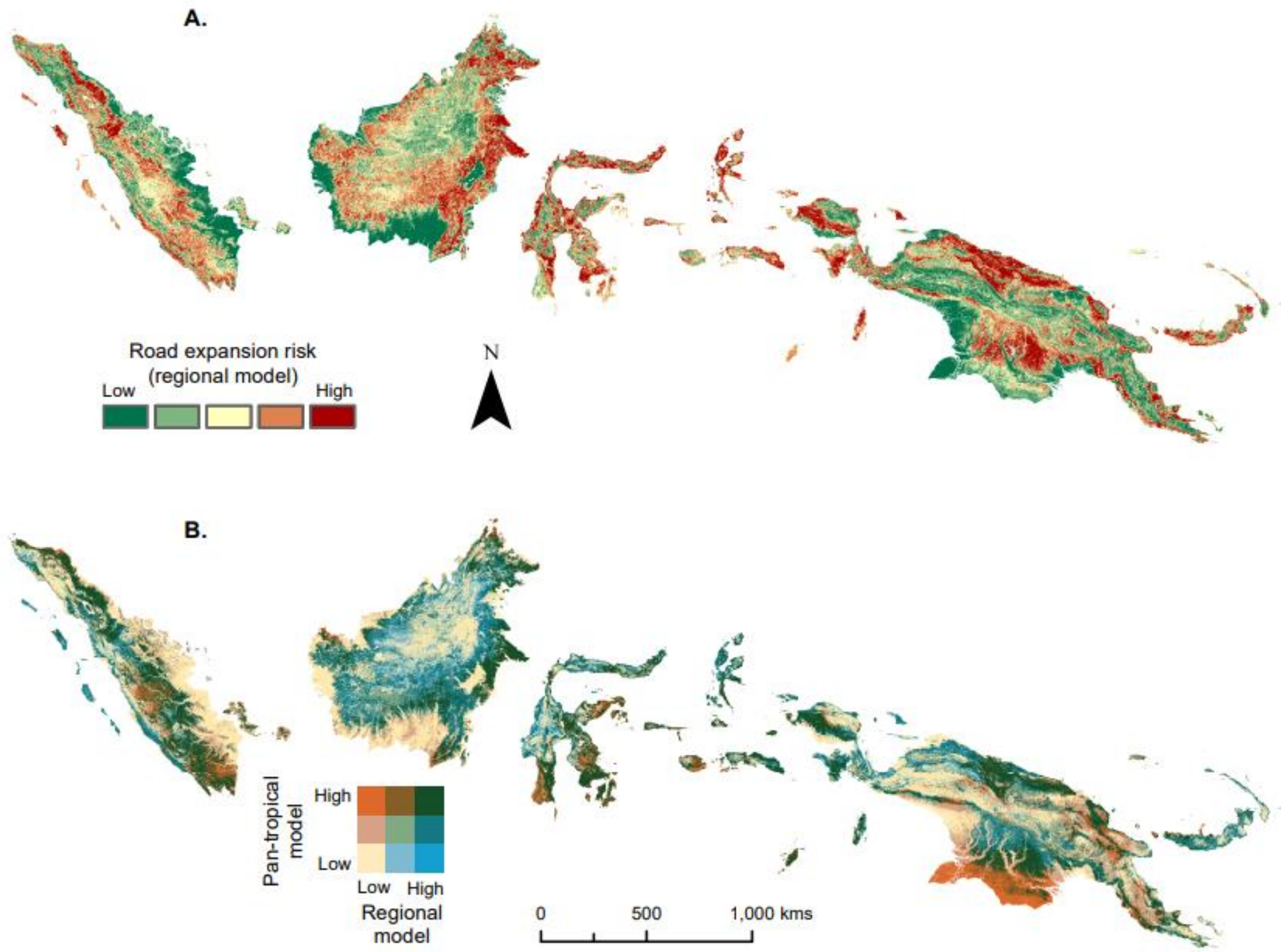

Fig. S4. Road-expansion risk from the region-specific model for the Asia-Pacific region and comparison to predictions from the pantropical model. For the comparison plot, low-medium-high thresholds are as follows: low = 0 – 0.45, medium = 0.45 – 0.55, high = 0.55 – 1.

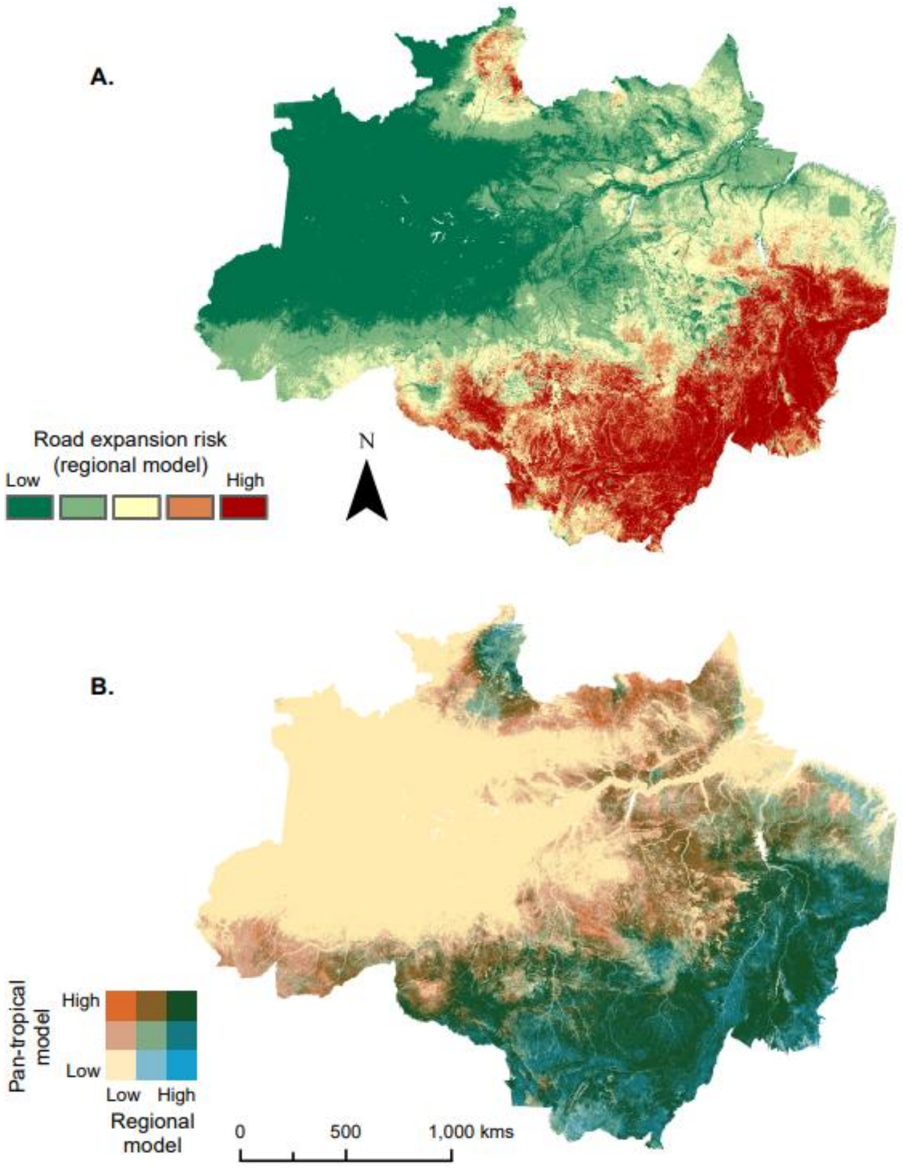

Fig. S5. Road-expansion risk from the region-specific model for the Brazilian Amazon and comparison to predictions from the pantropical model. For the comparison plot, low-medium-high thresholds are as follows: low = 0 – 0.45, medium = 0.45 – 0.55, high = 0.55 – 1.

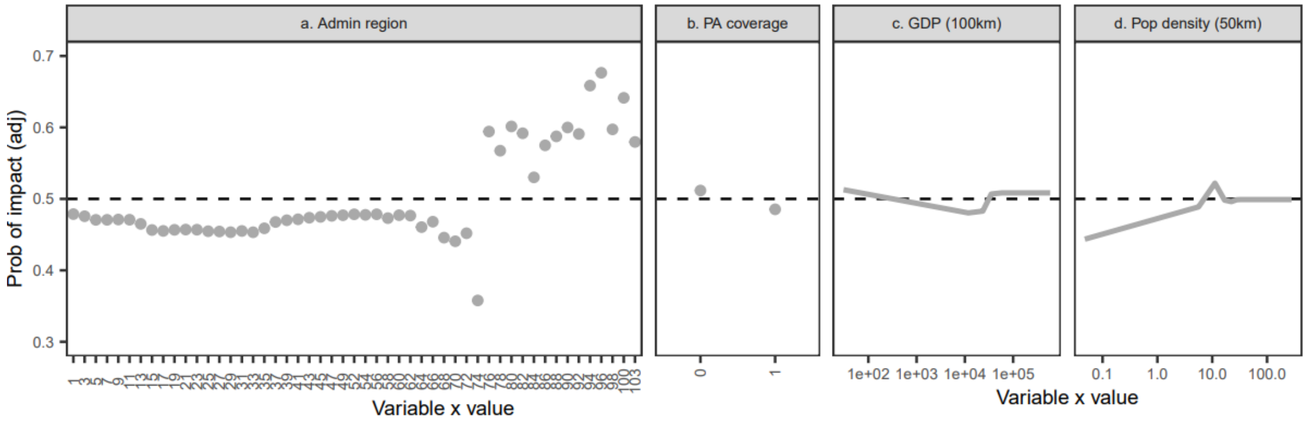

Fig. S6. Partial differential plots from the impact model for variables other than the road-expansion risk index.

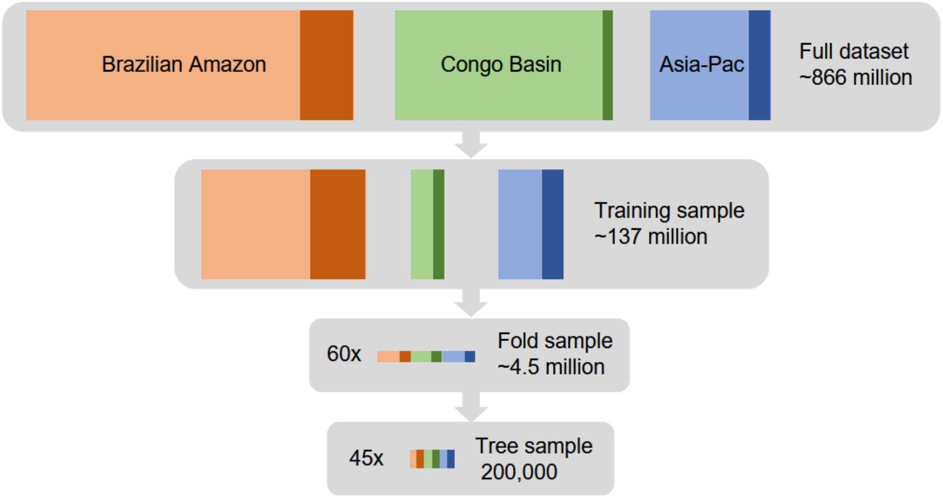

Fig. S7. Data sampling structure for random forest modelling. Dark coloured bars indicate the proportion of the data that has road presences, and the light coloured bars indicate the proportion with road absences.

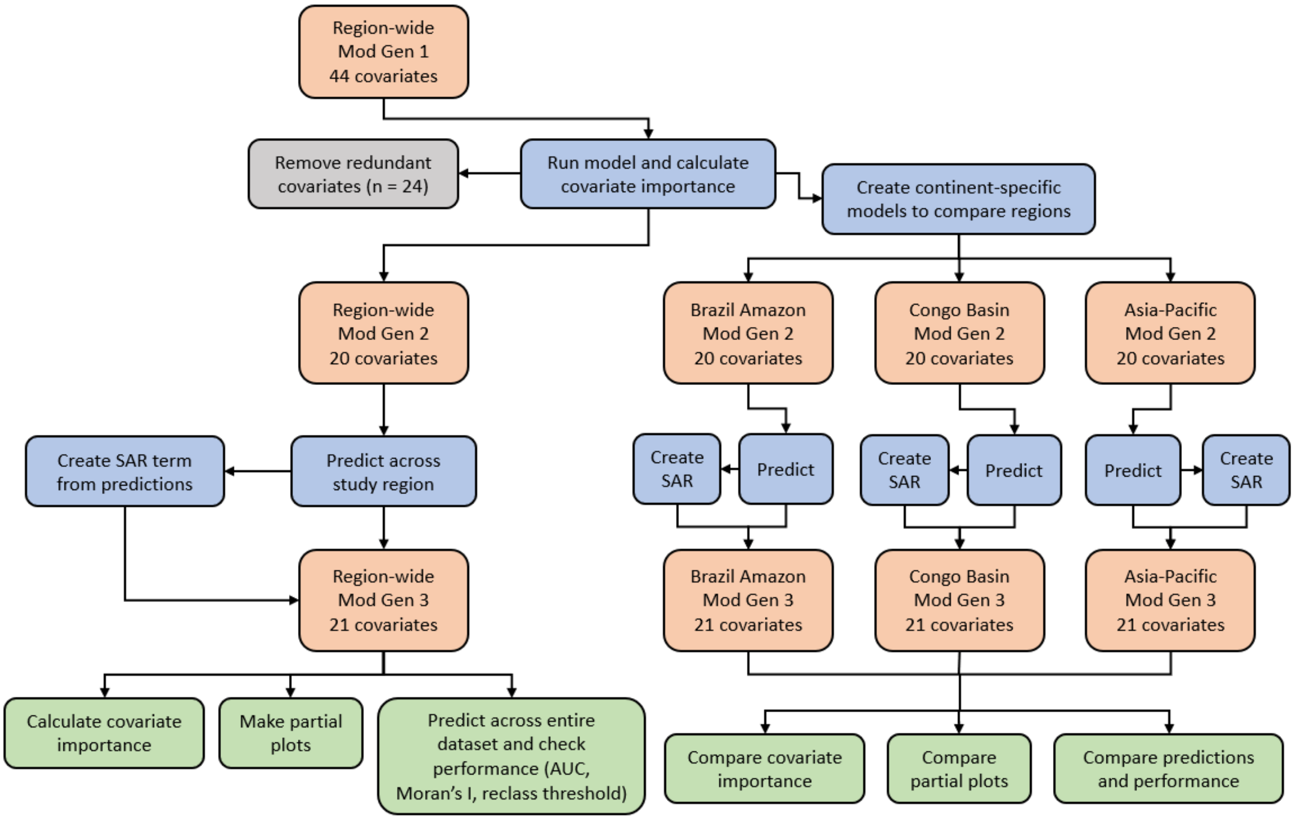

Fig. S8. Model generation structure for the region-wide model and continental-region-specific models. Models were trained on the training sample (~137 million observations) and performance metrics were calculated using the full dataset (~866 million observations).

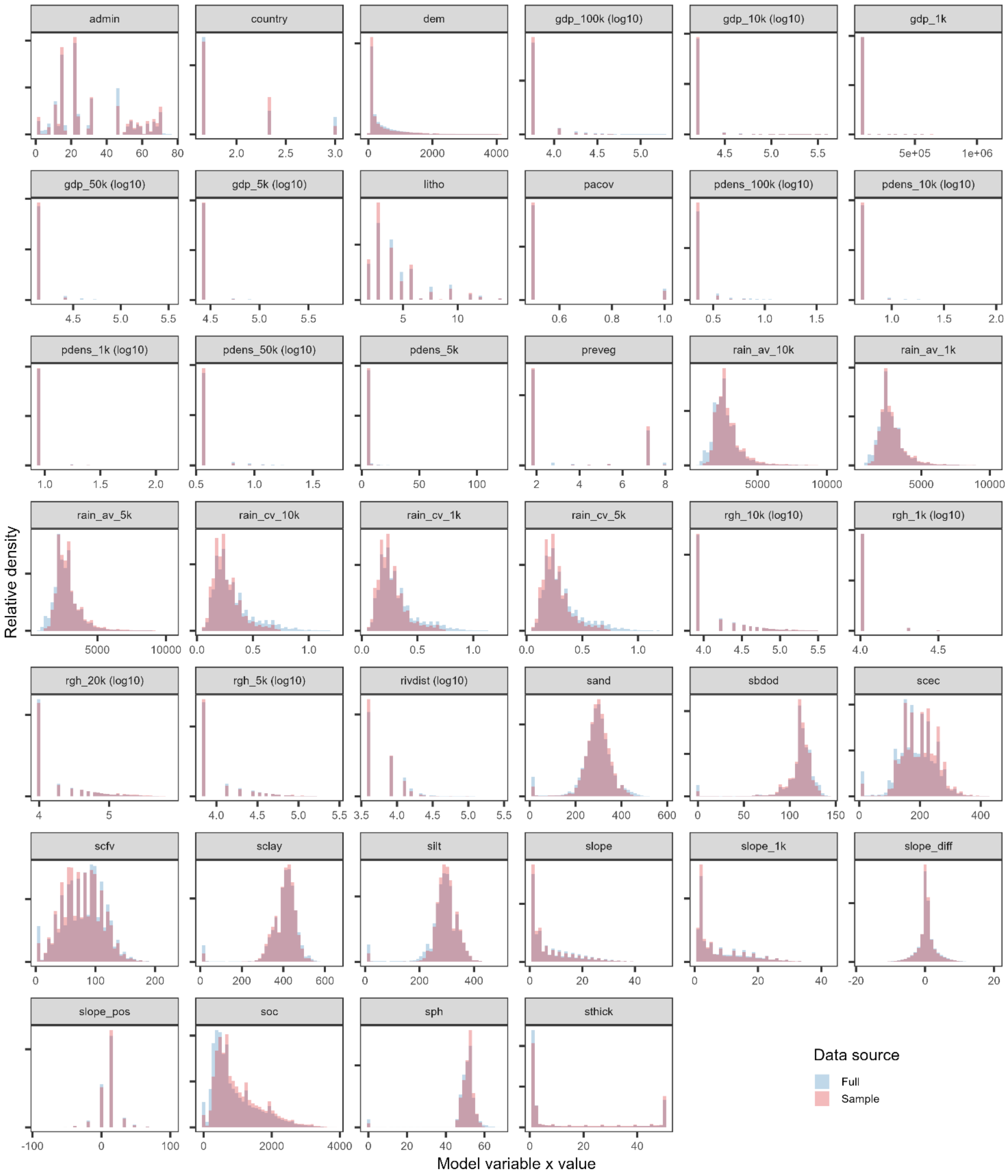

Fig S9. Comparison of value distributions for model variables between the full dataset (~866 million observations, blue) and the model training sample dataset (~137 million observations, red) for the Asia-Pacific region. Plots show overlaid histograms. Value distributions are near-identical for all variables.

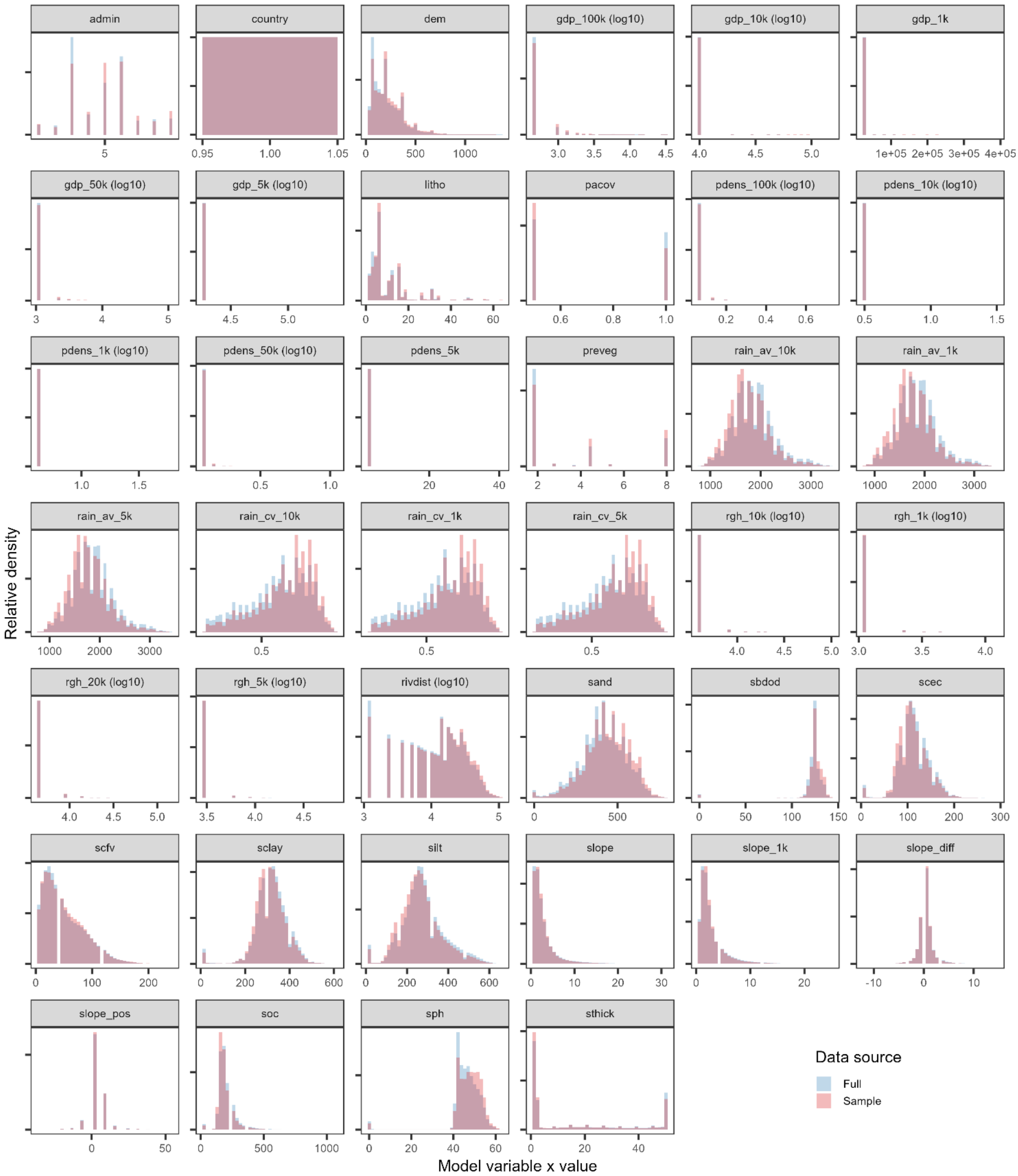

Fig S10. Comparison of value distributions for model variables between the full dataset (~866 million observations, blue) and the model training sample dataset (~137 million observations, red) for the Brazilian Amazon region. Plots show overlaid histograms. Value distributions are near-identical for all variables.

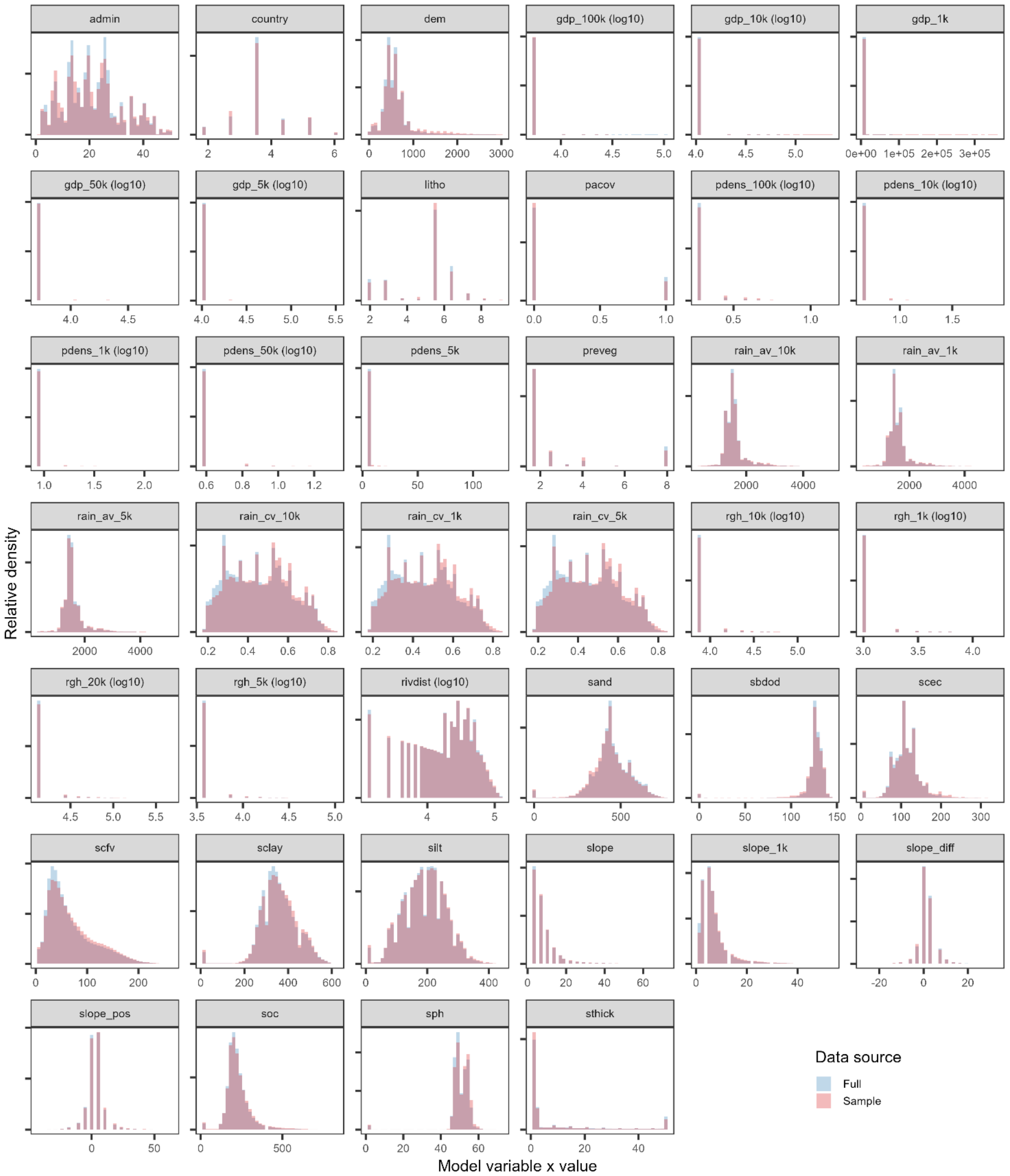

Fig S11. Comparison of value distributions for model variables between the full dataset (~866 million observations, blue) and the model training sample dataset (~137 million observations, red) for the Congo Basin region. Plots show overlaid histograms. Value distributions are near-identical for all variables.

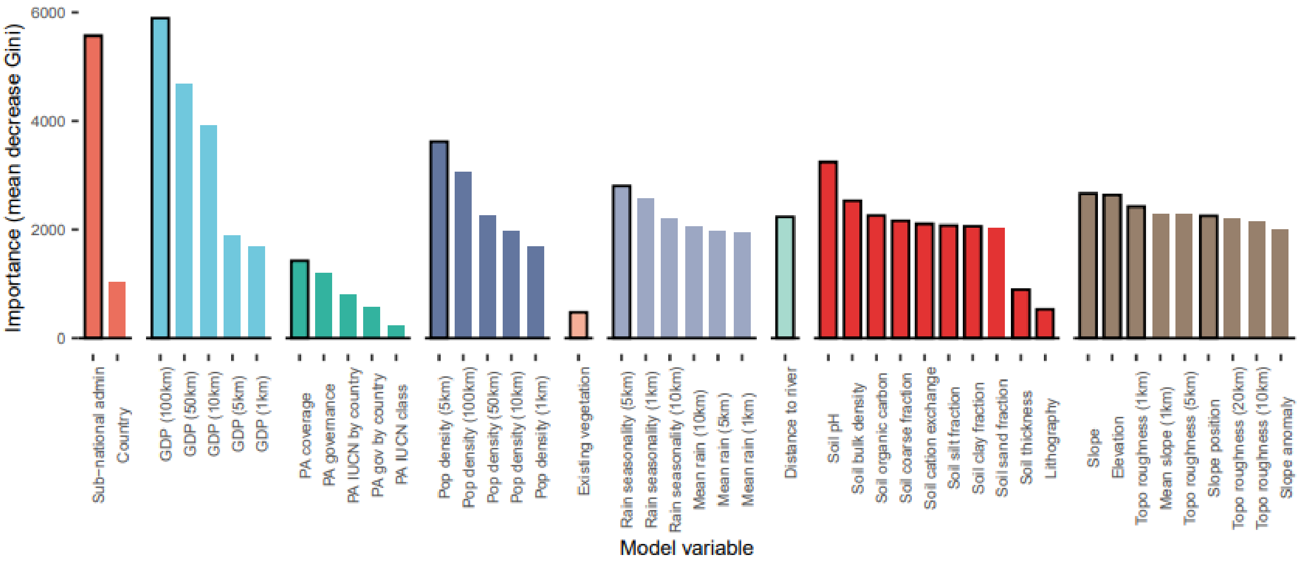

Fig. S12. Potential correlates of road construction and covariates retained in third-generation model. Columns with black outlines indicate the covariates that were retained in the second- and third-generation model, columns without outlines were not retained.

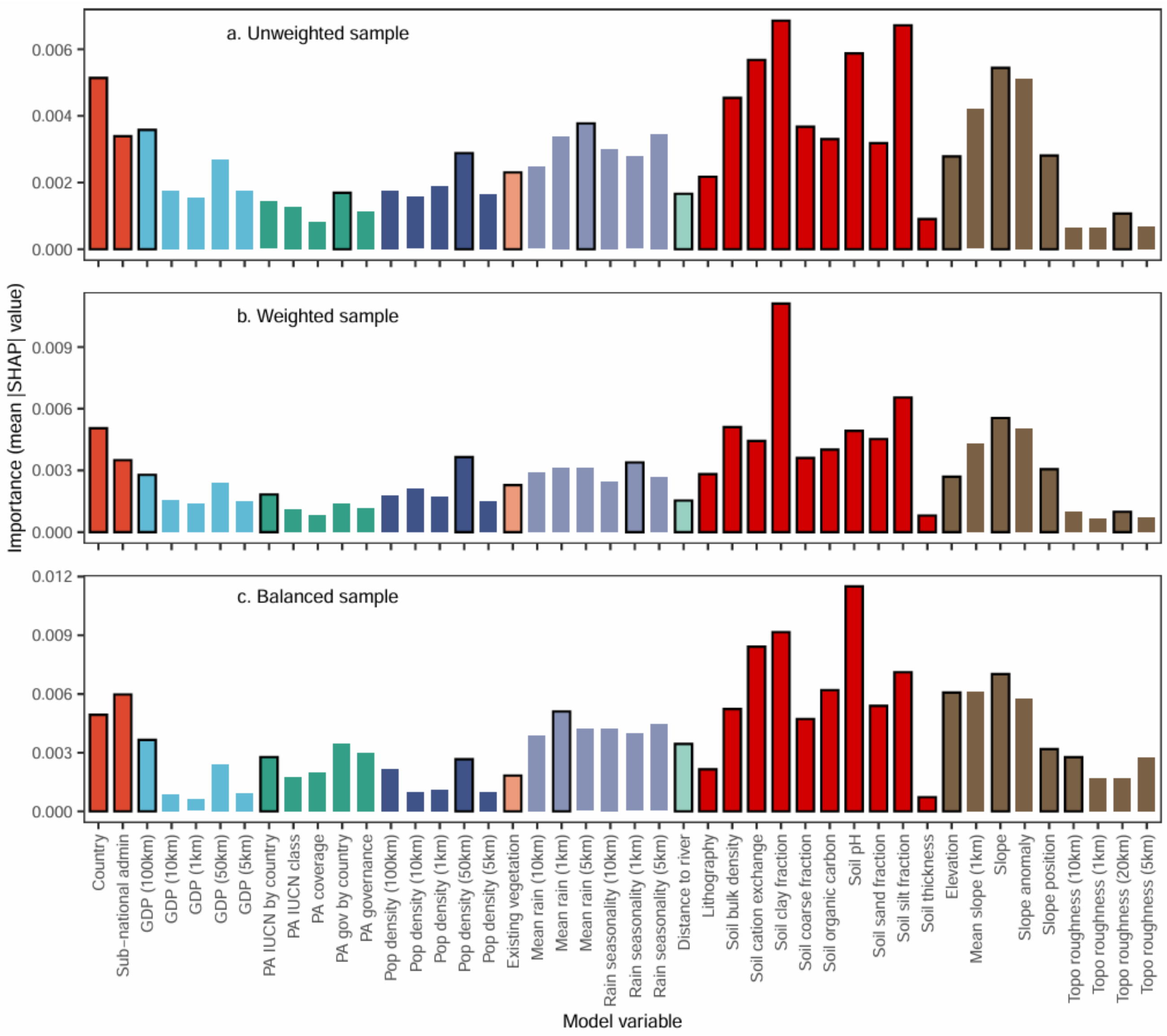

Fig S13. Comparisons of model variable importance (measured as mean absolute SHAP values) when using alternative model frameworks. SHAP values refers to the SHapley Additive exPlanations (SHAP) values. Balanced sample refers to the modelling framework where each tree was trained on an equal number of presences and absences (used in final models). Original prevalence refers to the modelling framework of training trees on a sample with proportions of presences and absences matching that of the full dataset (see Fig S7). Class weights refers to the model framework where trees were trained on the a sample at the original prevalence ratio but with class weights applied to counter the class imbalance.

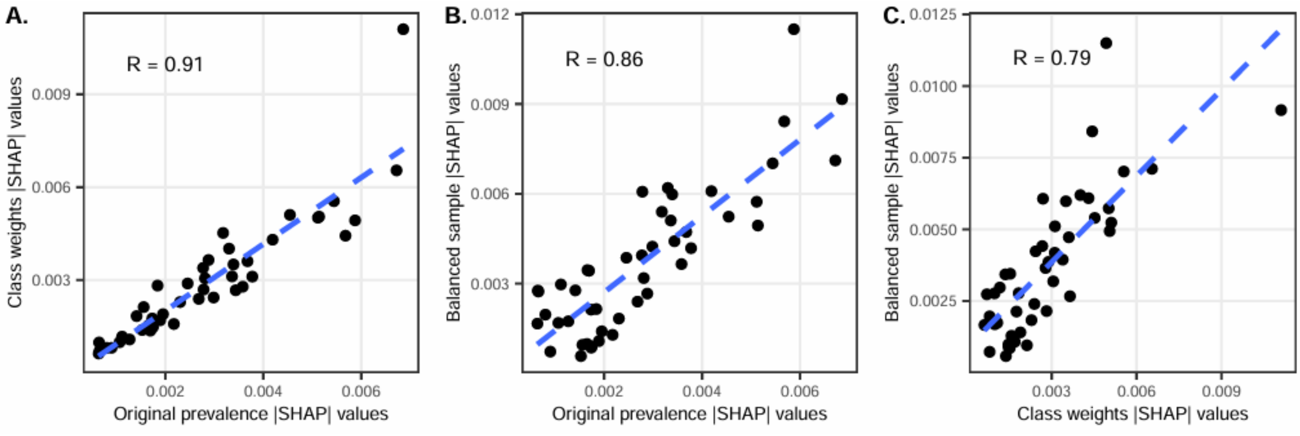

Fig S14. Correlations between model variable importance (measured as mean absolute SHAP values) among alternative model frameworks. Correlation (R values represent Pearson correlation coefficients) was high across all models demonstrating that variable importance was similar regardless of model framework. Balanced sample refers to the modelling framework where each tree was trained on an equal number of presences and absences (used in final models). Original prevalence refers to the modelling framework of training trees on a sample with proportions of presences and absences matching that of the full dataset (see Fig S7). Class weights refers to the model framework where trees were trained on the a sample at the original prevalence ratio but with class weights applied to counter the class imbalance.

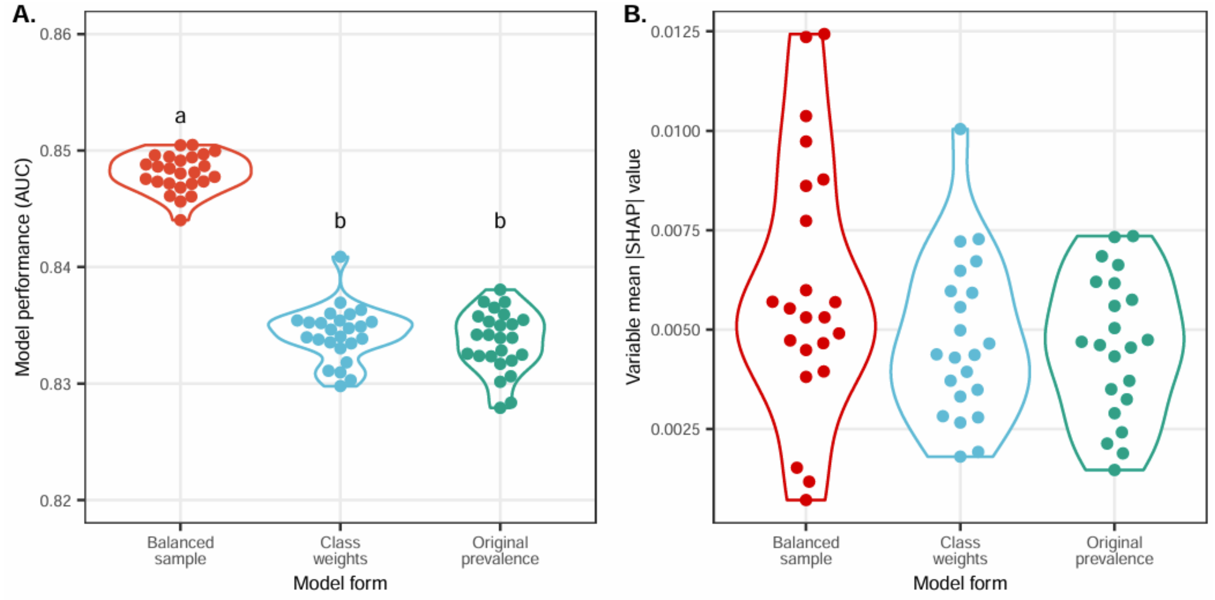

Fig S15. Model performance comparisons when using alternative data sampling and weighting formats. (A) comparison of model predictive performance (area under the receiver operator curve, AUC). (B) comparison of the explanatory value of model variables measured as the mean absolute values of the SHapley Additive exPlanations (SHAP) values. Inset letters in panel (A) refer to significant differences from Tukey post-hoc comparisons of the AUC values for each model. Both model performance and variable explanatory power where highest when using the balanced sample framework. Balanced sample refers to the modelling framework where each tree was trained on an equal number of presences and absences (used in final models). Original prevalence refers to the modelling framework of training trees on a sample with proportions of presences and absences matching that of the full dataset (see Fig S7). Class weights refers to the model framework where trees were trained on the a sample at the original prevalence ratio but with class weights applied to counter the class imbalance.

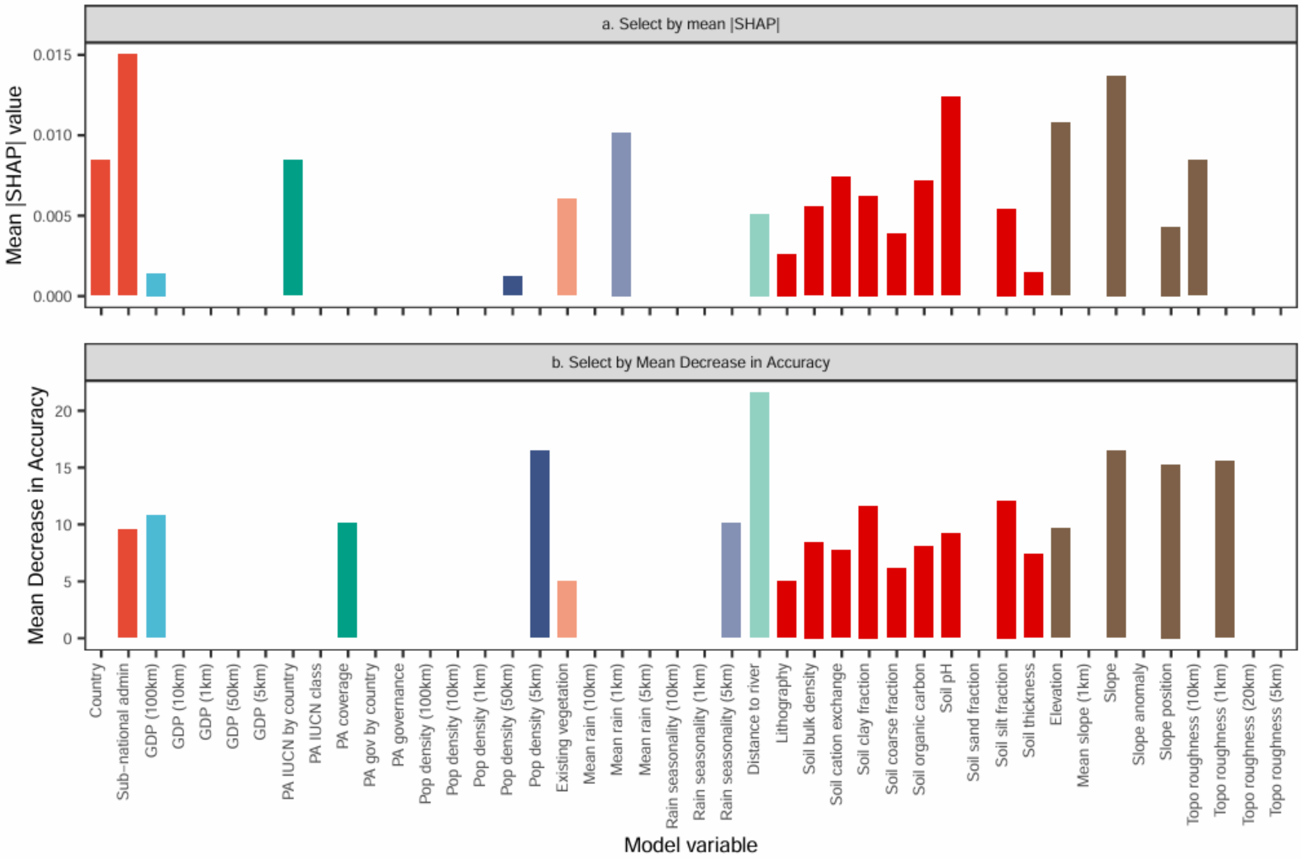

Fig S16. Comparison of variables retained in the final (third-generation) model when selecting by mean absolute SHAP values and mean decrease in permutation accuracy. SHAP values refers to the SHapley Additive exPlanations (SHAP) values.

Table S1. Potential correlates of road-expansion and deforestation. Model covariates were selected based on published studies and hypothesized relevance. Spatial data was sourced from high-quality published datasets and authoritative sources.

Variable	Type	Scale*	Rationale	Data source
Country	Admin	1ha	Governance has direct and indirect effects on road construction and human impacts (*1*), i.e. through expansionist development policy.	*2*
Sub-national admin region	Admin	1ha		
Population density	Soceco	1km<br>5km<br>10km<br>50km<br>100km	Population density has been shown to correlate with both road density (*3*) and human impacts (*4*).	*5*
Gross Domestic Product	Soceco	1km<br>5km<br>10km<br>50km<br>100km	As a raw measure of economic output, GDP is expected to be correlated with both infrastructure development and human impacts (*4*).	*6*
Protected area cover (%)	Admin	1ha	Protected areas can reduce rates of road construction (*7*) and forest loss (*8*), and this may differ between classes and governance types.	*10*
Protected area governance	Admin	1ha		
Protected area IUCN class	Admin	1ha		
PA IUCN class by country	Admin	1ha	Protected area effectiveness has been shown to differ significantly between countries (*9*).	
PA governance by country	Admin	1ha		
Distance to river	Enviro	1ha	Rivers may effect road planning and influence deforestation by providing access points (*8*).	Created from *11*
Pre-clearing vegetation	Enviro	1ha	We expect that different vegetation types may facilitate (i.e. grasslands) or constrain (i.e. wetlands) road construction.	*12*
Mean annual rainfall	Enviro	1km<br>5km<br>10km	Rainfall can influence road construction and degradation rates (*13*). Similarly it has been shown to influence deforestation rates (*7*).	*14*
Rainfall seasonality (Coefficient of Variation)	Enviro	1km<br>5km<br>10km		Created from *14*
Elevation	Enviro	1ha	Slope, elevation, and other topographic variables constrain road construction (*15*) and have been shown to reduce deforestation and other human impacts (*16*)	*17*
Slope	Enviro	1ha<br>1km		Created from *17*
Slope position	Enviro	1ha		
Slope anomaly**	Enviro	1ha		
Topographic roughness	Enviro	1km<br>5km<br>10km<br>20km		Created using *18*
Lithography	Enviro	1ha	Soil properties can influence road construction (*19*) and suitability for anthropogenic land uses (*20*), therefore influencing human impacts.	*21*
Soil sand fraction	Enviro	1ha		*22*
Soil clay fraction	Enviro	1ha		
Soil silt fraction	Enviro	1ha		
Soil bulk density	Enviro	1ha		
Soil coarse fraction	Enviro	1ha		
Soil cation exchange	Enviro	1ha		
Soil organic carbon	Enviro	1ha		
Soil pH	Enviro	1ha		
Soil thickness	Enviro	1ha		*23*

* all values apart from 1-ha indicate the diameter of focal windows over which variables were averaged.
** slope anomaly was calculated as the slope value for a the cell minus the 1km focal mean slope.

References

1. E. Y. Arima, P. Barreto, E. Araújo, B. Soares-Filho, Public policies can reduce tropical deforestation: Lessons and challenges from Brazil. Land use policy 41, 465–473 (2014).
2. GADM. https://gadm.org/data.html
3. D. R. Glover, J. L. Simon, The Effect of Population Density on Infrastructure: The Case of Road Building. Economic Development and Cultural Change 23, 453–468 (1975).
4. E. F. Nzunda, F. Midtgaard, Spatial relationship between deforestation and protected areas, accessibility, population density, GDP and other factors in mainland Tanzania. Forests, Trees and Livelihoods 26, 245–255 (2017).
5. A. Rose, J. McKee, K. Sims, E. Bright, A. Reith, M. Urban, LandScan Global 2020 [Data set]. Oak Ridge National Laboratory 10, 1524214 (2021).
6. M. Kummu, M. Taka, J. H. Guillaume, Gridded global datasets for gross domestic product and Human Development Index over 1990–2015. Scientific data 5, 1–15 (2018).
7. J. E. Engert, M. J. Campbell, J. E. Cinner, Y. Ishida, S. Sloan, J. Supriatna, M. Alamgir, J. Cislowski, W. F. Laurance, Ghost roads and the destruction of Asia-Pacific tropical forests. *Nature* 629, 370–375 (2024).
8. C. P. Barber, M. A. Cochrane, C. M. Souza Jr, W. F. Laurance, Roads, deforestation, and the mitigating effect of protected areas in the Amazon. Biological conservation 177, 203–209 (2014).
9. V. Graham, J. Geldmann, V. M. Adams, P. J. Negret, P. Sinovas, H.-C. Chang, Southeast Asian protected areas are effective in conserving forest cover and forest carbon stocks compared to unprotected areas. Scientific reports 11, 23760 (2021).
10. UNEP-WCMC, IUCN, Protected Planet: The World Database on Protected Areas (WDPA). https://www.protectedplanet.net/en (2023).
11. G. H. Allen, T. M. Pavelsky, Global extent of rivers and streams. Science 361, 585–588 (2018).
12. J. E. Engert, C. M. Souza, F. Kleinschroth, D. Juffe-Bignoli, S.P. Costa, J. Botelho Jr., Y. Ishida, I. Nursamsi, W.F. Laurance. Classifying and quantifying the impacts of frontier roads in tropical forests. Current Biology, 35(7), 1641-1648 (2025).
13. M. Alamgir, M. J. Campbell, S. Sloan, M. Goosem, G. R. Clements, M. I. Mahmoud, W. F. Laurance, Economic, Socio-Political and Environmental Risks of Road Development in the Tropics. Current Biology 27, R1130–R1140 (2017).
14. D. N. Karger, S. Lange, C. Hari, C. P. Reyer, N. E. Zimmermann, CHELSA-W5E5 v1. 0: W5E5 v1. 0 downscaled with CHELSA v2. 0. (2021).
15. P. Collier, M. Kirchberger, M. Söderbom, The Cost of Road Infrastructure in Low- and Middle-Income Countries. The World Bank Economic Review 30, 522–548 (2016).
16. D. L. Gaveau, M. Kshatriya, D. Sheil, S. Sloan, E. Molidena, A. Wijaya, S. Wich, M. Ancrenaz, M. Hansen, M. Broich, Reconciling forest conservation and logging in Indonesian Borneo. PloS one 8, e69887 (2013).
17. A. Jarvis, H. Reuter, A. Nelson, E. Guevara, Hole-filled SRTM for the globe version 3, from the CGIAR-CSI SRTM 90 m database (http://srtm.csi.cgiar.org, 2008).
18. J. S. Evans, J. Oakleaf, Geomorphometry & gradient metrics toolbox (ArcGIS 10.0, 2012).
19. S. M. Lim, D. Wijeyesekera, A. Lim, I. Bakar, Critical review of innovative soil road stabilization techniques. International Journal of Engineering and Advanced Technology 3, 204–211 (2014).
20. E. Barrios, Soil biota, ecosystem services and land productivity. Ecological economics 64, 269–285 (2007).
21. J. Hartmann, N. Moosdorf, The new global lithological map database GLiM: A representation of rock properties at the Earth surface. Geochemistry, Geophysics, Geosystems 13 (2012).
22. L. Poggio, L. M. De Sousa, N. H. Batjes, G. B. Heuvelink, B. Kempen, E. Ribeiro, D. Rossiter, SoilGrids 2.0: producing soil information for the globe with quantified spatial uncertainty. Soil 7, 217–240 (2021).
23. J. Pelletier, P. Broxton, P. Hazenberg, X. Zeng, P. Troch, G. Niu, Z. Williams, M. Brunke, D. Gochis, Global 1-km Gridded Thickness of Soil, Regolith, and Sedimentary Deposit Layers, ORNL DAAC, Oak Ridge, Tennessee, USA. (2016
